# Genetic Modification for Wheat Improvement: From Transgenesis to Genome Editing

**DOI:** 10.1155/2019/6216304

**Published:** 2019-03-10

**Authors:** Nikolai Borisjuk, Olena Kishchenko, Serik Eliby, Carly Schramm, Peter Anderson, Satyvaldy Jatayev, Akhylbek Kurishbayev, Yuri Shavrukov

**Affiliations:** ^1^School of Life Science, Huaiyin Normal University, Huaian, China; ^2^Institute of Cell Biology and Genetic Engineering, Kyiv, Ukraine; ^3^College of Science and Engineering, Biological Sciences, Flinders University, Bedford Park, SA, Australia; ^4^Faculty of Agronomy, S. Seifullin Kazakh AgroTechnical University, Astana, Kazakhstan

## Abstract

To feed the growing human population, global wheat yields should increase to approximately 5 tonnes per ha from the current 3.3 tonnes by 2050. To reach this goal, existing breeding practices must be complemented with new techniques built upon recent gains from wheat genome sequencing, and the accumulated knowledge of genetic determinants underlying the agricultural traits responsible for crop yield and quality. In this review we primarily focus on the tools and techniques available for accessing gene functions which lead to clear phenotypes in wheat. We provide a view of the development of wheat transformation techniques from a historical perspective, and summarize how techniques have been adapted to obtain gain-of-function phenotypes by gene overexpression, loss-of-function phenotypes by expressing antisense RNAs (RNA interference or RNAi), and most recently the manipulation of gene structure and expression using site-specific nucleases, such as CRISPR/Cas9, for genome editing. The review summarizes recent successes in the application of wheat genetic manipulation to increase yield, improve nutritional and health-promoting qualities in wheat, and enhance the crop's resistance to various biotic and abiotic stresses.

## 1. Introduction

Cereals are a key component of human diets, providing a significant proportion of the protein and calories consumed worldwide. While rice and maize dominate global cereal production, wheat is another vital crop consumed by humans, contributing to approximately 20% of our energy needs (calories) and 25% of our dietary protein. The Green Revolution of the 1970s achieved enormous yield gains via the introduction of disease resistant semidwarf high yielding wheat varieties developed by Dr. N.E.Borlaug and colleagues. Since that time, however, global wheat production has stagnated, and current trends show that yields will not be sufficient to meet growing market demands.

According to the United Nations' Food and Agriculture Organization (FAO), over 756 million tonnes of wheat grain was harvested from over 220 million ha of arable land in 2016/2017 (www.fao.org/faostat). Despite this, wheat lags behind other major cereals such as maize and rice, both in terms of yield, and the application of genomic tools for its improvement [1]. While the average worldwide yield grew almost 3-fold during the Green Revolution, driven by the expansion of irrigation, intensive use of fertilisers and advanced breeding [2]; the current average global wheat yield of ~3 tonnes per hectare is far below the crop's potential [3]. In order to feed the population of 9 billion people predicted for 2050, wheat yield should grow by over 60% while still maintaining and/or improving its nutritional characteristics [3, 4]. To achieve this goal without increasing the area of cultivated land, which is simply not available, emphasis must be concentrated on key traits related to plant productivity and adaptation to environmental challenges. A deficit in this key staple crop could present a serious threat to global food security, so improved molecular-based breeding and genetic engineering techniques are necessary to break through the current yield ceiling.

Existing modern breeding efforts now need to be complemented with advanced crop functional genomics, which can provide insights into the functioning of wheat genetic determinants. The available tools for wheat genetic modification provide the experimental means to functionally characterize genetic determinants by suppressing or enhancing gene activities. This knowledge can then be used for targeted improvements tailored to the specific needs of the diverse and changing environments in which wheat is grown across the world. This offers the potential to tackle yield gaps wherever they exist, for a variety of causes, enabling this global crop to finally reach its full potential.

## 2. Progress in Wheat Genetic Transformation

Bread wheat (*Triticum aestivum* L.), the most widespread of all wheat species, is an annual herb belonging to the family Gramineae or Poaceae. Wheat was domesticated around 8,000 years ago [29] and has since undergone hybridization and genome duplication events to generate its hexaploid genome (2n = 6x = 42, AABBDD), which is more than five times larger than the human genome. It was previously estimated that the genome of common wheat contained over 128,000 genes [30], with over 80% of the genome consisting of repetitive sequences of DNA [31]. However, more recent estimates suggested a total of 107,891 high-confidence genes with over 85% repetitive DNA sequences, representing a threefold redundancy due to its hexaploid genome [32].

Genetic transformation, the fundamental tool of genetic engineering, allows the introduction and expression of various genes of interest in the cells of living organisms, bypassing, when desirable, the barriers of sexual incompatibility that exist in nature. Despite the considerable efforts of the international research community, development of wheat genetic engineering lags behind that of the other key agricultural crops like rice and maize. This may be attributed to the genetic characteristics of wheat, including its very large (17,000 Mbp) and highly redundant complex genome, as well as the relative recalcitrance of most varieties to* in vitro* culture and regeneration (reviewed recently in [33]).

The first successful genetic transformation of common wheat was conducted at Florida University, USA [34], using biolistics and financed by a research grant from Monsanto. Scientists from Monsanto were also the first to report the generation of transgenic wheat using* Agrobacterium*-mediated transformation [35].

### 2.1. Wheat Transformation Methods

Presently, biolistics and* Agrobacterium*-mediated transformation using immature embryos as explants remain the main methods for genetic engineering of wheat. Each method has its own advantages and drawbacks (Table 1). The main advantages of* Agrobacterium* transformation are the relatively high ratio of single copy gene inserts and relative simplicity of the transformation procedure. In contrast, biolistics offer benefits in their capacity to transform organelles and deliver RNA, proteins, nanoparticles, dyes, and complexes into cells. Utilization of linear Minimal Expression Cassettes (MECs) in biolistic transformation enables the production of plants carrying much simpler patterns of transgene integration compared to plasmid bombardment, with a higher proportion of single copy inserts [36–38].

In addition, biolistic delivery of MEC simplifies the simultaneous cotransformation of several genes and, in contrast to* Agrobacterium*, does not introduce vector backbone DNA or repetitive border sequences flanking the T-DNA into the transformed plant cells. In experiments with the wheat cv. ЕМ12, transformation with MECs instead of plasmids improved transformation frequency (TF) almost threefold from 0.4% tо 1.1% [39]. A simplified method for DNA/gold coating was described by Ismagul et al. [38] for the high-throughput biolistic production of single copy transgenic wheat utilizing diluted MECs. This method involves the application of PM solution (42% PEG 2000 and 560 mM MgCl_2_) instead of the spermidine and CaCl_2_ used in the standard Bio-Rad procedure.

Biolistics allow for the transfer into wheat of relatively large DNA fragments. Partier et al. [40] conducted successful biolistic transformation of wheat with a 53 Кb linear cassette which contained a 44 Kb fragment of an* Arabidopsis* gene flanked by selection and reporter genes. The intact cassette was detected in Т_1_ and Т_2_ generation plants. The main disadvantages of both* Agrobacterium* and biolistic transformation methods in wheat are genotype dependency, and the requirement for lengthy periods of aseptic tissue culture.

### 2.2. Wheat Transformation Frequencies

Until recently, the TF for most tested genotypes of common wheat remained quite low at a level mostly below 5% [8]. Many research groups use model wheat varieties such as Bobwhite SH98-26 and Fielder in their experiments due to their amenability to transformation via published protocols [8, 9]. The cultivar Bobwhite SH98-26 was among 129 sister lines made from crosses of cultivars Aurora//Kalyan/Bluebird 3/Woodpecker at CIMMYT, and selected for its high TF of over 70% by biolistic transformation [9]. Such high transformation efficiency is yet to be reproduced by other researchers, and although the reason for this remains unclear, it is possible that not all of the finer details of the transformation protocol could be described in the report. The success rate of this high TF may be explained by the particular hybrid genotype used, or simply the advanced skills and experience of the technicians at CIMMYT. Since publishing [9], A. Pellegrineschi has been employed by two big Biotech companies, Pioneer Hi Breed and Du Pont, which indirectly confirms that he has developed a reliable wheat transformation protocol that allowed him to produce the published results. The authors of the review are therefore not sceptical; however the high TF presented in [9] must be reproduced by other researchers in future if this protocol is to be widely adopted. The cultivar Fielder, which is the model variety in* Agrobacterium* transformation, was preselected by the researchers of the Japan Tobacco Company, where the detailed protocol “PureWheat” was developed [8] that allows TFs of 40–90%. In this protocol, positive selection through the application of phosphomannose isomerase (PMI), which converts mannose-6-phosphate into fructose-6-phosphate, was found to facilitate a relatively high TF (20%) in biolistic transformation of the spring wheat line UC703 [41].

### 2.3. Agrobacterium-Mediated Transformation

In recent years several groups have reported efficient* Agrobacterium* transformation of a number of wheat cultivars [8, 42–44]. Hensel et al. [43] developed a protocol to transform the model genotype Bobwhite SH98-26 with TFs up to 15%. In the experiments of Richardson et al. [42] using the PureWheat technology with the cv. Fielder, selection on 5-10 mg/l of phosphinothricin resulted in a TF of 41.0%. Without selective pressure, the TF was only around 2.3%. Similar results were obtained for Chinese commercial cultivars of wheat by Wang et al. [44], who demonstrated TF of 37.7% for cv. СВ037, 22.7% for cv. Kenong 199, and 45.3% for the model cv. Fielder. It was shown by Ishida et al. [8] that centrifugation of immature embryos before infection with* Agrobacterium* was one of the critical requirements for successful transformation, while heat shock, contrary to findings in other cereals, was not efficient. At present*, Agrobacterium* strains EHA101, EHA105 [8], AGL0, AGL1 [43, 45], GV3101 [46], C58C1 [47, 48], and LBA4404 [49] are the most popular in wheat transformation.

Transformation using mature wheat embryos is currently characterized by relatively low TFs. Wang et al. [48] transformed longitudinally cut mature embryos and observed TFs of 0.06%, 0.67%, and 0.89% for the cultivars Bobwhite, Yumai 66, and Lunxuan 208, respectively. Aadel et al. [50] found that, with the application of 200 *μ*М acetosyringone, the TFs for the genotypes Rajae and Amal were 0.66% and 1.00%. The protocol of Medvecka and Harwood [51], using Bobwhite SH98-56, allows production of transformants at a TF of 2.2%. More information and a simplified protocol can be found in [52].

In contrast to* in vitro *methods, the* in planta* approach has the potential to overcome the problem of high genotype dependency seen with the existing transformation methods. There have been several publications on the* in planta* production of transgenic wheat, most involving the direct injection of* Agrobacterium*. Supartana et al. [49] reported agrobacterial transformation of wheat cv. Shiranekomugi using seeds soaked overnight in water. Transformation was achieved by double piercing the area where a shoot would later emerge with a needle dipped into* Agrobacterium *inoculum. This method used the* Agrobacterium *strains LBA4404 and an M-21 mutant strain, and no tissue culture steps were used at any stage. The plants obtained were analysed for antibiotic resistance, and by PCR, Southern hybridization and plasmid rescue to confirm their transgenic status. Zhao et al. [53] produced* Agrobacterium-*mediated transgenic wheat by adding inoculum to an incision made at the base of wheat seedlings. Their tissue culture-free method was reportedly successful in transferring a powdery mildew-resistance gene, with a TF of up to 9.82%. Similarly, in the experiments of Razzaq et al. [54] with the wheat cv. GA-2002, the apical meristems of imbibed wheat seeds were wounded and inoculated with the* Agrobacterium* strain LBA4404 containing the binary vector pBI121 (35S-GUS, pNOS-NPTII). The kanamycin resistant plants produced were analysed by PCR and GUS histochemical staining of the embryos. Risacher et al. [20] developed an efficient semi-*in planta* transformation protocol (US patent 7803988 B2, 2010) that involves* in planta* agrobacterial infection of immature embryos within developing seeds. Spikes of the spring wheat line NB_1_ were harvested 16 to 18 days after anthesis and* Agrobacterium* injected into the base of each spikelet. The spikes, with their flag leaves still attached, were incubated in low light for 2 to 3 days before embryos were isolated and cultured* in vitro*. The protocol achieved an average TF of 5% with 30-50% of plants carrying a single transgene insertion.

The practice of dipping floral buds in a suspension of* Agrobacterium* (Floral dip) is a routine and highly efficient method of transformation in* Arabidopsis, *but it is rarely successful in other plant species. However, Zale et al. [21] were able to generate three independent transgenic lines of the spring wheat line, Crocus, by utilizing the floral dip approach. The transformants were studied thoroughly at the molecular level for three to six generations. In their most recent publication, Hamada et al. [22] trialled a biolistic method for* in planta *transformation. They found that bombarding the exposed shoot apical meristems of the wheat cultivars Fielder and Haruyokoi, using 0.6 *μ*m gold particles and 1350 psi pressure, resulted in the integration of the GFP reporter gene into the germline cells in 62% of regenerated plants (transformants), including possible chimeric individuals. The full potential of the wheat* in planta* procedures published to date is yet to be fully realized.

Microspore transformation using immature pollen grains is a method that generates doubled haploid homozygous wheat plants in a single generation. The great advantage of this technique is that it by-passes the several years required by other transformation methods to develop true-breeding transformant lines. Current protocols for microspore-based transgenic wheat production through electroporation, biolistics, and* Agrobacterium*-mediated transformation are presented in [55–57]. Various wheat genotypes have been used as donors. Express, Chris, Farnum, Hollis, Louise, Perigee, and WestBred 926 have all performed successfully in microspore transformation. Four genes were targeted for microspore transformation, including *β*-glucuronidase (GUS),* uidA*; bialaphos resistance,* Bar*;* Trichoderma harzianum* endochitinase gene,* ThEn42*; and* Bacillus subtilis* 1,4-*β*-xylanase gene [55–57]. In general,* Agrobacterium-*mediated microspore transformation produces the best outcome, generating stable homozygous plants and fewer chimeric plants. Given the value of doubled haploid lines in simplifying and accelerating wheat breeding, it is likely that microspore methods will be optimized in the future.

Recent advances in high-throughput sequencing technologies have allowed for more detailed information to be obtained about the composition and fate of transgenes introduced into plant cells through* Agrobacterium*-mediated transformation. In one report, close to 0.4% of transgenic* Arabidopsis* lines examined contained insertions of* Agrobacterium *chromosomal DNA [16]. Singer et al. [58] showed, for the first time, the presence in transgenic plant cells of the extra-chromosomal circular T-DNA that they named “T-circles”. In rice, 26% of 331 analysed transgenic lines contained the transposon Tn5393, which was transferred from the* Agrobacterium* strain LBA4404 [59]. This transposon was not detected in the strains EHA105 and GV3101. These data reflect the importance of detailed molecular analysis of transgenic lines, and careful selection of the appropriate agrobacterial strains for transformation. A better understanding of the biological mechanisms of the transfer and integration of transgenes during bacterial infection will also permit the creation of new more efficient strains of* Agrobacterium* [5, 60] or utilization of bacterial species that are not pathogenic in nature [61].

### 2.4. Optimization of Tissue Culture Conditions

Tissue culture conditions are important factors that impact the TF of all plants, including wheat. Below we present information reflecting the current trends in wheat tissue culture that might be of interest to researchers working in the field. Changing the composition of the macrosalts in the growth medium can positively affect the TF. Callus induction on 6x DSEM medium with an increased concentration of ammonium nitrate (62.56 mM) as the sole nitrogen source, resulted in the sevenfold improvement of TF during biolistic transformation of the elite wheat cv. Superb [62]. The authors point out that this modification of the medium brought about an increase in the number of somatic embryoids and possibly also reduced stress during the bombardment of the cells due to the elevated osmolarity. Ishida et al. [8] identified some critical points in the transformation process and developed a detailed protocol for* Agrobacterium*-mediated transformation for both immature and mature wheat embryos from amenable genotypes. The authors however did not discuss any work on media improvements.

Antioxidants such as ascorbic acid, glutathione, lipoic acid, selenite, and cysteine were found to decrease necrosis and darkening of the tissues, improving plant regeneration and TF during genetic transformation [63]. Lipoic acid at 25–50 *μ*М led to a twofold improvement in agrobacterial transformation of the wheat cv. Bobwhite [64].

Arabinogalactan-proteins at a concentration of 5 mg/l improved regeneration of bread (cv. Ikizce-96) and durum (cv. Mirzabey) wheat from 77.77% and 72.11%, to 94.86% and 89.73%, respectively [65]. Kumar et al. [66] were able to achieve close to 100% plant regeneration from calli induced from wheat mature and immature embryos on 2.0 mg/l picloram, using a regeneration medium with 0.1 mg/l 2,4-D, 5.0 mg/l zeatin, and 15 mg/l CuSO_4_. Miroshnichenko et al. [67] developed a protocol for the regeneration of einkorn wheat (*Т*.* monococcum* L.), in which a combination of 3.0 mg/l dicamba, 50.0 mg/l daminozide (an inhibitor of ethylene synthesis), and 0.25 mg/l thidiazuron in the regeneration medium was the most efficient. The authors presume that this protocol may be useful for other wheat species as well.

### 2.5. Transient Transformation

Transient transformation with the use of reporter genes such as GUS [68] and GFP [69] is helpful for optimization of transformation conditions, as well as the analysis of promoters and protein expression. Transgene expression that is tightly targeted to specific tissues and developmental stages is often desired for directed modification of morphogenic traits. It can also be beneficial for avoiding feedback mechanisms, transgene silencing, or other unforeseen effects that can arise from constitutive transgene expression. Transient assays using protoplasts can also facilitate efficient analysis of gene regulatory mechanisms through cotransformation of enhancer/represser elements and promoter-reporter gene constructs [11]. Viral induced gene silencing (VIGS) offers a fast and rapid transient assay for silencing of gene expression. VIGS can be achieved through a simple vacuum-aided cocultivation of germinated wheat seeds with* Agrobacterium* carrying an appropriate VIGS construct [70]. However, results from VIGS are not comparable to results from stable transformation experiments, but this does not mean that the VIGS findings are false or of no value. Like RNAi, VIGS findings are useful for increasing our understanding of gene function and may lead to the development of a strategy that is more successful than simple constitutive transgene overexpression.

### 2.6. Future Directions: Gene Stacking and Plant Artificial Chromosomes

Gene stacking or pyramiding (defined as the stacking of multiple transgenes at a single chromosomal location) greatly expands the potential for genetic engineering of traits in wheat [71]. Applications of this technology include modifying complex and multigenic traits, or inserting entire biosynthetic pathways, with integration at a single locus. This significantly simplifies the subsequent breeding process. Alternatively, for disease resistance traits, transgene stacks can aid in generating broad spectrum resistance to multiple threats, or more long-lasting defence to a single pathogen via genes with differing modes-of-action. Construction of a transgene stack is most often achieved using type II restriction enzymes in the Golden-Gate cloning system, or by Gibson Assembly. The integration of longer DNA fragments carries certain risks, however, such as reduced TF, fragmentation leading to only partial integration, and transgene silencing if promoters or other regulatory elements are used repeatedly. The magnitude of these risks differs according to the nature of the vectors or transformation cassettes, the transgene products, and the recipient genotype. Much of the more recent work involving stacked transgenes uses site-specific nucleases so that the transgene stack can be directed to a favourable locus in the genome. Site-specific nucleases will be covered in a later section of this review.

Plant artificial chromosomes or minichromosomes were also developed for multigene transfer [72]. The main advantage is that they generate a new genetic locus that segregates independently of endogenous chromosomes, thus lessening the disruption of existing genomic regions. Minichromosomes have been developed in mammalian cells through a process named “Telomere-mediated chromosome truncation” that targets highly conserved telomeric repeat sequences. Analogous sequences have been identified in* Arabidopsis*, but progress in developing plant minichromosomes still lags behind the work in mammalian cells. Minichromosomes have the potential to act as “super-vector platforms” for the organization and expression of foreign genes and may even be designed with Cre/lox recombination sites to accept the introduction of new genes. This presents the option to “add on” additional alleles or genetic elements at a later date. Currently, the reliable transmission of minichromosomes to the progeny of primary transformants presents the greatest barrier to the application of this technology. However, initial work has suggested that wheat's allopolyploid nature makes it more tolerant to chromosomal truncation than that of diploid species, making this a very promising future prospect for wheat genetic engineering [73].

## 3. Manipulating Agronomic Traits by Transgene Expression in Wheat

Built on the progress made on techniques for genetic manipulation of wheat, overexpression of endogenous and foreign genes has tremendously enriched our understanding of the functionality of numerous genes, contributing to the generation of many new and improved agronomically important traits. This improved understanding of gene function has led to the development of specific promoters to drive gene expression in response to environmental stimuli and/or in a tissue, organ, or developmental stage-specific manner [74, 75]. Various signals for directing expressed proteins to particular cellular compartments have also been used [76]. Also, the field received another major stimulus through the use of transcription factors that function as major regulators of gene networks involved in numerous metabolic/physiological pathways. Transcription factors are often highly conserved between different plant species, so the information obtained from studying model plants like* Arabidopsis* or rice can be applied to other plants such as wheat [77–79].

In pursuit of improved crop performance, wheat genetic manipulations made by transgenic gene expression have targeted all major agronomic traits including yield, grain quality, and tolerance to abiotic and biotic stresses. The last 10 years have been characterized by acceleration in this field and an increasing number of publications. In this review we will focus primarily on attempts to improve yield and grain quality, the two areas that have not been sufficiently reviewed by other authors. For readers interested in improving wheat stress responses, we refer to recent comprehensive reviews on the topics of pathogen resistance [80–82] and abiotic stress tolerance [83, 84], as well as a recent review of transgenic wheat engineered with various genes of agronomic importance [33].

### 3.1. Yield

Yield is determined by the number and size of grains produced by the crop. The major genes controlling yield-related traits in wheat have been identified by genetic and genomics techniques, as reported in recent reviews [85–87].

Grain size (GS) has long been a focus of wheat selection and modern breeding [88]. Progress in comparative wheat genomics has led to the identification of* TaGW2 *[89], a wheat homolog of a rice gene that negatively influences GS through regulation of cell division within the spikelet hull [90]. The first experiments aimed at downregulating* TaGW2* in wheat have shown some controversial results. Bednarek et al. [91] demonstrated that expression of a specific RNAi to suppress three* TaGW2 *homologs A, B, and D, of bread wheat cv. Recital, led to a decrease in grain size and weight. In contrast, Hong et al. [92], in a similar series of experiments, demonstrated a significant* increase* in the grain width and weight of a Chinese bread wheat cv. Shi 4185 that is typically characterized by small grains. Such differences in the phenotypes obtained in these two studies may be explained by a cultivar-specific reaction to experimental intervention, and/or by the application of different transformation protocols, as has been previously reported for barley [93].

Plant growth and productivity to a large extent relies on the uptake of carbon, nitrogen, and phosphorus. Ribulose-1,5-bisphosphate carboxylase/oxygenase (RuBisCo), the most abundant protein on Earth, is the major enzyme assimilating atmospheric carbon in the form of CO_2_ into organic compounds in photosynthetic organisms. However, the enzyme has relatively low efficiency and a slow turnover rate [94]. Despite significant progress in characterizing the enzyme's properties [95] and improving RuBisCo efficiency in a range of plant species [96, 97], little success has been achieved so far in commercial crops, including wheat. Another area for optimizing carbon assimilation in crops is to introduce components of the C_4_ photosynthetic apparatus into C_3_ plants like wheat and rice. C_4_ plants, such as maize, display definite advantages over plants with C_3_ photosynthesis, especially under high temperature and limited water conditions [98]. These advantages rely on a number of specific enzymes associated with RuBisCo, which provide higher photosynthetic efficiency and CO_2_ assimilation for the C_4_ plants. Transferring genes encoding the C_4_-specific enzymes, phosphoenolpyruvate carboxylase (PEPC), and pyruvate orthophosphate dikinase (PPDK) has led to promising results in rice [99] and wheat [100, 101]. As an example, Zhang et al. [101] generated transgenic wheat plants overexpressing PEPC and PPDK both separately and simultaneously in the same transgenic lines. The authors showed a positive synergistic effect on wheat photosynthetic characteristics and yield in the latter design. The follow-up study on wheat expressing maize PEPC demonstrated that, in addition to increased yield, the transgenic lines had improved drought tolerance linked to elevated expression of proteins involved in photosynthesis and protein metabolism [102].

The maize gene encoding the transcription factor Dof1, known to upregulate the expression of PEPC, was introduced into wheat by* Agrobacterium*-mediated transformation [103]. Expression of* ZmDof1* under the control of the light-inducible RuBisCo promoter, led to increased biomass and yield components in transgenic wheat, while constitutive expression resulted in the downregulation of photosynthetic genes and a corresponding negative impact on crop productivity.

The Nuclear Factor Y (NF-Y) transcription factors are recognized as important regulators of many plant developmental and physiological processes [104]. NF-Ys are composed of protein subunits from three distinct transcription factor families (NF-YA, NF-YB, and NF-YC), with each of them represented by multiple members [105].* TaNFY-A-B1*, a low-nitrogen- and low-phosphorus-inducible NF-YA transcription factor, was overexpressed in wheat. This led to a significant increase in nitrogen and phosphorus uptake and grain yield in a field experiment [106]. The authors suggested that increased nutrient uptake resulted from stimulated root development and upregulation of both nitrate and phosphate transporters in the roots of the transgenic wheat. Our own work showed the positive role of the second wheat gene,* TaNF-YB4*, in wheat grain yield [107]. Constitutive overexpression of the gene under the control of the maize ubiquitin promoter in transgenic wheat cv. Gladius, resulted in the development of significantly more spikes, and a 20–30% increase in grain yield compared to the untransformed control plants.

Overexpression of another wheat transcription factor,* TaNAC2-5A,* which plays a role in nitrogen signalling, enhanced root growth and the nitrate influx rate, consequently increasing the root's ability to acquire nitrate from the soil. Transgenic wheat lines revealed higher grain yield and higher nitrogen accumulation in aerial parts of the plant, which was subsequently allocated to grains [108].

Recently, a US research group reported on the expression of a rice gene encoding a soluble starch synthase gene (*OsSS-I*) with increased heat stability. This lead to a significant, 21-34%, yield increase in T_2_ and T_3_ generations of transgenic wheat under heat stress conditions [109]. The expression of* OsSS-I* also prolonged the duration of the photosynthetic growth period in bread wheat. Similarly, overexpression of an endogenous gene coding for the chloroplastic glutamine synthase gene (*TaGS2*) in wheat led to prolonged leaf photosynthesis, and an increased rate of nitrogen remobilization into grains, which translated to higher spike number, grain number per spike, and total yield [110]. Taken together, these reports on the modulation of carbon and nitrogen pathways once again illustrate the tight link between nitrogen assimilation and carbon metabolism [111] and resulting crop productivity.

### 3.2. Grain Quality

Grain is the harvested part of the wheat plant and its nutritional and health properties are determined by its biochemical composition. Starch, making up 55–75% of total dry grain weight, and storage protein 10–15%, are the main reserves of the wheat seed. Therefore, starch and protein greatly affect the quality of the products made from wheat flour. Along with optimized starch and protein, an adequate level of essential elements like iron, zinc, calcium, phosphorus, and antioxidants is also essential for healthy and balanced wheat products. Many of those quality traits have been addressed in recent years by means of transgenic interventions.

A number of studies have focused on the biosynthetic pathways and composition of starch, as reviewed by Sonnewald and Kossmann [112] and more recently by Kumar et al. [113]. Based on the insights gained, a number of biotechnological interventions have been undertaken in order to both increase the amount of starch, and modulate its quality, with mixed outcomes. In an attempt to increase the level of precursors for starch synthesis in wheat, Weichert et al. [114] overexpressed the barley sucrose transporter gene (*HvSUT1*), which led to enhanced sucrose uptake and protein content in wheat grains, but no significant modification to starch levels. Expression of an optimized maize ADP-glucose pyrophosphorylase (*ZmAGPase*) resulted in elevated yield and enhanced photosynthetic rates in the transgenic wheat lines [115]. Downregulation of the transcription factor* TaRSR1*, a wheat homolog of Rice Starch Regulator (*OsRSR1*), which was shown to negatively regulate the gene expression of some starch synthesis-related enzymes in wheat grains [116], resulted in a significant 30% increase in starch content, and also a ~20% increase in yield in terms of 1000-kernel weight [117]. The increases in starch and yield were underpinned by the marked induction of expression of many key-genes in sugar metabolism and starch biosynthesis.

Starch consumer quality depends mostly on the ratio of amylose to amylopectin, the two main macromolecules forming starch. Starch with increased amylose has attracted much interest because of its contribution to resistant starch (RS) in food, which confers beneficial effects on human health. There is evidence that RS can provide protection from several health conditions such as diabetes, obesity, and cardiovascular diseases [118]. A number of experiments focused on downregulation of starch branching enzymes, SBEIIa, and SBEIIb, which led to substantially increased amylose levels in wheat [119, 120]. High amylose starch has demonstrated positive health-related effects in rats [119] and more recently in a study involving obesity in humans [121].

Nitrogen is not only the most important plant nutrient contributing to crop yield, but also plays a significant role in defining the accumulation and, to some extent, the composition of storage protein in wheat grains [122]. Nitrogen is supplied to the grain by two major pathways: remobilization from the canopy (leaves and stems), and root uptake from the soil. In their experiments, Zhao et al. [123] identified a novel wheat gene,* TaNAC-S*, a member of the NAC transcription factor family, that showed decreased expression during leaf senescence but significant expression in a stay-green phenotype. Transgenic overexpression of the gene in wheat resulted in delayed leaf senescence and increased protein concentration in grains, while the crop's biomass and grain yield remained unaffected. Another group of Chinese researchers overexpressed a tobacco nitrate reductase gene (*NtNR*) in two commercial winter wheat cultivars, ND146 and JM6358, following* Agrobacterium*-mediated transformation [124]. Constitutive overexpression of the gene remarkably enhanced foliar nitrate reductase activity and resulted in significantly augmented seed protein content and 1000-grain weight in the majority of the T_1_ offspring analysed.

The main component of wheat storage proteins, gluten, primarily determines the viscoelastic properties of wheat dough [125]. Glutens consist of gliadins and glutenins, which together comprise 70-80% of the total flour protein. Therefore, genes encoding different classes of storage proteins have been targeted in efforts to improve both the nutritional value and bread-making quality of wheat. In fact, the genes coding for high-molecular glutenin subunits (HMW-GS) were among the first introduced into wheat [126–129], with the aim of improving dough functions following the first reports of a method for wheat transformation. Specifically, introduction of the subunits 1Ax1 and 1Dx5 into several common wheat cultivars by genetic transformation has demonstrated the potential of the transgenes to enhance dough quality to various extents [130–132]. In follow-up studies, the HMW-GS genes were introduced into selected wheat cultivars, mostly via the easily transformed cv. Bobwhite and then transferred into selected elite commercial varieties. Improvements in dough properties were obtained, demonstrating the feasibility of utilizing transgenic lines in wheat breeding programs [133, 134].

In contrast to HMW glutenins, which form complex polymer structures and are strongly correlated to dough elasticity, gliadins are monomeric components that contribute mainly to the extensibility and viscosity of the dough [135]. Based on their electrophoretic mobility, gliadins are grouped into three structural types: *α*-, *γ*-, and *ω*-gliadins, each encoded by tightly-linked multigene clusters. The interest in genetic modification of gliadins has been stimulated, not only by their contribution to dough quality, but also because they include the majority of immunogenic epitopes related to immune conditions such as wheat-dependent exercise-induced anaphylaxis (WDEIA) and coeliac disease [136, 137].

Iron and zinc are essential micronutrients for human nutrition. According to the World Health Organization, over a billion people suffer from iron-deficiency anaemia, and Zn-deficiency is estimated to be associated with an annual death rate of almost half a million children under the age of five. Modern elite wheat cultivars usually contain suboptimal quantities of micronutrients [138], and because most of it is accumulated in the outer husk, aleurone, and embryo, the micronutrients are lost during milling and polishing [139]. Another problem is that phytic acid, a major antinutritional factor for iron and zinc uptake in the human digestive tract, is codeposited with the minerals in aleurone storage vacuoles.

Biofortification, the enhancing of crop nutritional quality, is considered a promising approach to alleviate micronutrient deficiency. Transgenic studies have made significant headway in the development of strategies aimed at improving the available content of micronutrients such as iron in wheat grains. Plants store iron primarily in the form of ferritin structures accumulated mainly in nongreen plastids, etioplasts, and amyloplasts. Expression of a gene coding for an* Aspergillus niger* phytase, a phytic acid degrading enzyme targeted to the wheat aleurone [140], and endogenous [141] or soybean [142] ferritin genes in wheat endosperm, were the first successful attempts to transgenically biofortify wheat grains with iron. Recently, Connorton et al. [143] have isolated, characterized, and overexpressed two wheat Vacuolar Iron Transporter (*TaVIT*) genes under the control of an endosperm-specific promoter in wheat and barley. They reported that the introduction of one of the genes,* TaVIT2*, resulted in a greater than twofold increase in iron in the flour prepared from transgenic wheat grains without other detected changes.

## 4. RNA Interference Applications in Wheat

RNA interference (RNAi) is a common regulatory mechanism of gene expression in eukaryotic cells that has become a powerful tool for functional gene analysis and the engineering of novel phenotypes. The technique is based on the expression of antisense or hairpin RNAi constructs, or other forms of short interfering RNA molecules to direct posttranscriptional gene silencing in a sequence specific manner.

The vernalisation gene,* TaVRN2,* was the first wheat gene to be targeted by RNAi in transgenic wheat plants [144].* TaVRN1* mRNA in transgenic plants was reduced by 60%, which led to much earlier flowering. In another study, Loukoianov et al. [145] suppressed expression of* TaVRN1* by up to 80%, delaying flowering time by 14 to 19 days and increasing the number of leaves relative to the nontransgenic controls. These two breakthrough studies provided essential evidence for understanding the molecular mechanisms of flowering timing and vernalisation requirements in wheat, which may assist in diversifying the environments in which wheat can be grown.

The application of RNAi has made a solid contribution to manipulating wheat grain size [146–148] and quality [119, 149–151]. For example, Alterbach and Allen [152] used RNAi silencing to suppress the expression of *ω*-gliadins associated with WDEIA in the US wheat cv. Butte 86. They later demonstrated that transgenic wheat lines deficient in *ω*-gliadins had no changes in patterns of other grain proteins, and even showed increased protein stability with improved dough properties under various growth conditions [153]. Similarly, Gil-Humanes et al. [154] downregulated the *γ*-gliadin genes in the wheat cv. Bobwhite and then transferred the trait into three commercial wheat cultivars by conventional crossing.

The reduction of *γ*-gliadins in wheat grains was compensated by an increased amount of glutenins, which led to stronger dough with better over-mixing-resistance. In a recent comprehensive study, Barro et al. [149] used a combination of seven RNAi expressing plasmids to selectively target *α*-, *γ*-, and *ω*-gliadins, and Low Molecular Weight (LMW) glutenins in the wheat cv. Bobwhite, with the goal to reduce gluten epitopes related to coeliac disease (CD). The protein analyses showed that three RNAi plasmid combinations resulted in total absence of CD epitopes from the most immunogenic *α*- and *ω*-gliadins in the transgenic wheat lines. These very promising results pave the way to developing wheat varieties with nonallergenic properties.

However, the major application of RNAi technology for wheat has been in pathogen and pest control using virus- and host-induced gene silencing platforms [155]. A recent strategy, referred to as host-induced gene silencing (HIGS), has been developed to silence pest or pathogen genes by plant-mediated RNAi during their feeding or attempted infection, thereby reducing disease levels. This strategy relies on the host-plant's ability to produce interfering RNA molecules complementary to targeted pest/pathogen genes. These molecules are then transferred to the invader, causing silencing of the targeted gene. In wheat, HIGS has been most widely applied to control fungal and insect diseases. As an example, silencing of fungal glucanosyltransferase genes (*GTF1* and* GTF2*), and the virulence effector gene* Avra10,* affects wheat resistance to the powdery mildew fungus* Blumeria graminis* [156]. Three hairpin RNAi constructs corresponding to different regions of the* Fusarium graminearum* chitin synthase gene (*Chs3b*) were found to silence* Chs3b* in transgenic* F*.* graminearum* strains. Coexpression of these three RNAi constructs in two independent elite wheat cultivar transgenic lines, conferred high levels of stable and durable resistance to both* Fusarium* head blight and* Fusarium* seedling blight [157]. Stable transgenic wheat plants carrying an RNAi hairpin construct against the *β*-1, 3-glucan synthase gene* FcGls1* of* F. culmorum,* or a triple combination of* FcGls1* with mitogen-activated protein kinase (*FcFmk1*) and chitin synthase V myosin-motor domain (*FcChsV*), also showed enhanced resistance in leaf and spike inoculation assays under greenhouse and near-field conditions, respectively [158].

In other examples of RNAi, targeting the myosin-5 gene (*FaMyo5*) from* F. asiaticum* provided disease resistance in wheat [159]. In further examples, wheat transformed with a vector expressing a double-stranded RNA, targeting the mitogen-activated protein kinase kinase gene (*PsFUZ7*) from* Puccinia striiformis* f. sp.* tritici* exhibited strong resistance to stripe rust [160]. Transgenic wheat plants expressing siRNAs targeting the catalytic subunit protein kinase A gene from* P. striiformis* f. sp.* Tritici,* displayed high levels of stable and durable resistance throughout the T_3_ to T_4_ generations [161]. Stable expression of hairpin RNAi constructs with a sequence homologous to mitogen-activated protein‐kinase from* P. triticina* (*PtMAPK1*), or a cyclophilin (*PtCYC1*) encoding gene, in susceptible wheat plants showed efficient silencing of the corresponding genes in the interacting fungus, resulting in disease resistance throughout the T_2_ generation [162].

The grain aphid (*Sitobion avenae*) chitin synthase 1 gene (*CHS1*) was also targeted with HIGS. After feeding on the representative T_3_ transgenic wheat lines,* CHS1* expression levels in grain aphid decreased by half, and both total and moulting aphid numbers reduced significantly [163]. Other target genes used for the control of grain aphids by RNAi in transgenic wheat were lipase maturation factor-like2 gene from pea aphid* Acyrthosiphon pisum *[164], carboxylesterase gene [165],* Hpa1* [166], and a gene encoding a salivary sheath protein [167]. Feeding on these transgenic plants resulted in significant reductions in survival and reproduction of aphids. It seems that the scope of RNAi-induced pest and disease resistance is as broad as the number of different pathogen and pest species that infect and cause damage to wheat.

## 5. Site-Specific Nucleases for Targeted Genome Modifications in Wheat

Targeted genome engineering using nucleases, such as zinc finger nucleases (ZFNs) and TAL effector nucleases (TALENs), were developed in the late 20^th^ century as innovative tools to generate mutations at specific genetic loci [168]. Nuclease-based mutagenesis relies on induced site-specific DNA double-strand breaks (DSBs), which are either repaired by error-prone nonhomologous end joining (NHEJ), or high-fidelity homologous recombination (HR). The former often results in insertions or deletions (InDels) at the cleavage site leading to loss-of-function gene knockouts, whereas the latter leads to precise genome modification. In 2012, the field of eukaryotic genome editing was revolutionized by the introduction of CRISPR/Cas9 (bacterial Clustered Regularly Interspaced Short Palindromic Repeats) technology [169]. This technology confers targeted gene mutagenesis by a Cas9 nuclease that is guided by small RNAs (sgRNAs) to the target gene through base pairing. This is in contrast to the DNA-recognition protein domain that must be specifically tailored for each DNA target in the case of ZFNs and TALENs. Because of its universality and operational simplicity compared to ZFNs and TALEN genome editing systems, CRISPR/Cas9 has rapidly superseded these earlier editing systems and been adopted by the majority of the scientific community [170, 171].

In wheat, the principal applicability of CRISPR/Cas9 was demonstrated in protoplasts and suspension cultures, where multiple genes were successfully targeted in the year following the publication of the original CRISPR/Cas9 principle [172–174]. Original methods for plant genome editing rely on the delivery of plasmids carrying cassettes for the coexpression of Cas9 and sgRNA, either by* Agrobacterium* or particle bombardment. For gene editing in wheat, a Cas9 protein containing one or more signals for nuclear localization is expressed from a codon optimized gene under the control of RNA polymerase II promoters such as CaMV35S or ZmUbi, while the sgRNA is usually expressed from a polymerase III promoter (most commonly, rice or wheat U6 and U3 promoters).

One of the additional advantages of the CRISPR/Cas9 system is its potential for multiplexing, i.e., the simultaneous targeting of several genes with a single molecular construct. Multiple sgRNAs can be introduced either as separate transcription units, or in polycistronic form [175]. In bread wheat, editing has been reported in PEG-transfected protoplasts [172, 173, 176–178], electroporated microspores [179], and cell suspension cultures transformed by* Agrobacterium* [174]. Edited wheat plants have been regenerated from immature embryos, immature embryo-derived callus, or shoot apical meristems transformed via particle bombardment [176, 177, 180–184] or* Agrobacterium* [185, 186].

Recently, protocols for DNA-free editing of wheat by delivering* in vitro* transcripts, or ribonucleoprotein complexes (RNPs) of CRISPR/Cas9 by particle bombardment, have been developed [176, 177]. The authors claim that these methods not only eliminate random integration of the CRISPR/Cas9 coding DNA elements into the targeted genome, but also reduce off-target effects. Thus, these advances allow for the production of completely transgene-free mutants in bread wheat with high precision. The main limitation of these transgene-free protocols is the lack of selection in the transformation and regeneration process. Another optimized delivery system has been developed by Gil-Humanes et al. [177]. Here the authors used replicated vectors based on the wheat dwarf virus (Geminiviridae) for cereal genome engineering. It was shown that, due to increased copy number of the system components, virus-derived replicons increase gene targeting efficiency greater than 10-fold in wheat callus cells and protoplasts, compared to the nonreplicating control. The virus-based CRISPR/Cas9 system also promoted multiplexed gene targeted integration in different loci of the polyploid wheat genome by homologous recombination.

Since the advent of the principle of RNA guided nuclease genome editing, a number of additional tools for genome modifications and functional genomics studies have been developed [187]. The DNA binding ability of Cas9 and Cas12 has been used to develop tools for various applications, such as transcriptional regulation and fluorescence-based imaging of specific chromosomal loci in plant genomes. Another nuclease, Cas13, has been applied to degrade mRNAs and combat viral RNA replication [188]. Orthologues of Cas9 found in other bacterial species such as* Neisseria meningitides *[189],* Staphylococcus aureus *[190], and* Campylobacter jejuni *[191] have different and more complex Protospacer adjacent motif (PAM) sequences. These PAM sequences function in their native bacterial hosts to direct the CRISPR/Cas9 complex to the target sequence and not to the CRISPR/Cas9 locus. Although the use of these orthologous Cas9 proteins does limit the available target sequences for genome editing, it also reduces off-target edits. Most recently, systems for targeted base editing in wheat have been established by fusing a cytidine deaminase [181] or adenosine deaminase [192], to the Cas9 nickase for C/G to T/A or A/T to G/C conversion. In these systems, the efficiency of base editing was enhanced by using a Cas9-based nickase instead of an inactive Cas9. As an example, the base editor Cas9-APOBEC3A was used to edit* TaMTL* (*MATRILINEAL*) encoding a sperm-specific phospholipase [182]. Loss of function of* MTL* triggers haploid induction in maize [193]. Ten base-edited wheat mutants with* TaMTL* knock-out were identified at a frequency of 16.7%, with three being homozygous for all six alleles without InDels. Functional analysis of wheat mutants with* TaMTL* knock-out is still to be completed. Other nucleases with similar editing functions to that of Cas9 have been identified [194]. Most notably, Cpf1 possesses both DNase and RNase activity and cleaves DNA to generate four to five bp 5′-overhangs, potentially enhancing insertion of DNA sequences by homologous recombination. The Cpf1-based editing system has been successfully applied in plants, but not in wheat at this stage [195, 196].

Although many agriculturally important traits of wheat have been targeted by genome editing, some of the main ones include the following: (i) resistance/tolerance to biotic and abiotic stresses, (ii) yield and grain quality, and (iii) male sterility.

(i) The first successful experiment using the CRISPR/Cas9 system in wheat was editing of* TaMLO*, a powdery mildew-resistance locus. Powdery mildew diseases caused by* Blumeria graminis* f. sp.* tritici* result in significant wheat yield losses, and knock-out of the* TaMLO* leads to disease resistance. The mutation frequency of* TaMLO* in protoplasts was 28.5% [172]. Further, Wang et al. [180] described editing of the* TaMLO-A1* allele by the CRISPR/Cas9 system and simultaneous editing of three homoeoalleles of* TaMLO* in hexaploid bread wheat using the TALEN nuclease. The mutation frequency of regenerated* TaMLO*-edited wheat (5.6%) was similar for both editing methods. More recently, Zhang et al. [174] used CRISPR/Cas9 technology to generate* Taedr1* wheat lines by simultaneous knock-down of the three homologs of wheat* TaEDR1*, a negative regulator of powdery mildew resistance. The mutated plants were resistant to powdery mildew and did not show mildew-induced cell death [174].

The lipoxygenase genes,* TaLpx1* and* TaLox2,* attracted attention as potential subjects for gene editing in relation to resistance to* Fusarium*, one of the most devastating fungal diseases in wheat. Lipoxygenases hydrolyze polyunsaturated fatty acids and initiate biosynthesis of oxylipins, playing a role in the activation of jasmonic acid-mediated defence responses in plants. Silencing of the* TaLpx*-1 gene has resulted in resistance to* Fusarium graminearum* in wheat [197].* TaLpx1* and* TaLox2* genes were edited in protoplasts with a mutation frequency of 9% and 45%, respectively [173, 183]. Wheat plants with mutated* TaLOX2* were obtained with a frequency of 9.5%, of which homozygous mutants accounted for 44.7% [198].

The CRISPR/Cas9 system was also used for editing a wheat homolog of* TaCer9* (*ECERIFERUM9*) with the goal to improve drought tolerance and water use efficiency [199]. Mutation of the* AtCer9 *gene in* A. thaliana*, encoding an E3 ubiquitin ligase, causes elevated amounts of 18-carbon-length cutin monomers and very-long-chain free fatty acids (C24, C26) in cuticular wax, both of which are associated with elevated cuticle membrane thickness and drought tolerance [200].

The Cas9 nickase fused to a human cytidine deaminase, APOBEC3A, was used to produce herbicide resistant wheat plants through editing of* TaALS* [182]. ALS encodes acetolactate synthase, the first enzyme of the branched-chain amino acid biosynthesis. Amino acid substitutions in* TaALS* confer resistance to the sulfonylurea class of herbicides. Wheat plants with a mutated* TaALS* gene were obtained in high frequency (22.5%, 27/120). Among them, two plants had six alleles simultaneously edited and were nicosulfuron resistant.

(ii) With the aim of enhancing grain size and yield, several genes have been edited by the CRISPR/Cas9 system:* TaGASR7* [176, 184, 198],* TaGW2* [198, 199, 201], and* TaDEP1* [198].* TaGASR7*, a member of the* Snakin/GASA* gene family, has been associated with grain length in wheat. A CRISPR/Cas9 vector designed to target* TaGASR7* was delivered by particle bombardment into shoot apical meristems. Eleven (5.2%) of the 210 bombarded plants carried mutant alleles, and the mutations of three (1.4%) of these were inherited in the next generation [184]. Transiently expressing the CRISPR/Cas9 DNA and using the CRISPR/Cas9 RNP-mediated method were also highly effective for* TaGASR7* editing [176, 198]. The* TaGW2* gene encodes a previously unknown RING-type E3 ubiquitin ligase that was reported to be a negative regulator of grain size and thousand grain weight in wheat [198, 199, 201]. Recent studies detailing the functionality of the allelic* TaGW2 *genes through genome-specific knockouts [201], showed that the* TaGW2* gene in wheat acts by regulating the gibberellin hormone biosynthesis pathway [202], principally confirming the parallel functions of these genes in rice and wheat. T_1_ plants carrying knock-out mutations in all three copies of the* TaGW2 *gene (genotype* aabbdd*) showed significantly increased thousand grain weight (27.7%), grain area (17.0%), grain width (10.9%), and grain length (6.1%) compared to the wild-type cultivar [183].

Another gene editing target is* DENSE AND ERECT PANICLE 1* (*DEP1*), which encodes a G protein gamma-subunit in rice that is involved in the regulation of erect panicles, number of grains per panicle, nitrogen uptake, and stress tolerance through the G protein signalling pathway [203]. Zhang et al. [198] applied the CRISPR/Cas9 system to target* TaDEP1* and* TaNAC2* editing in wheat. CRISPR/Cas9-induced mutation of* TaNAC2* (a member of a plant specific transcription factor family) and* TaDEP1* in wheat plants was successful in 2% of cases [198]. One possible outcome of the loss of* TaNAC2* function could be increased size of grain and changes in stress responses.* TaPIN1* (*PINFORMED1*) was edited using CRISPR/Cas9 technology with a frequency of 1% [198]. The plant specific* PIN* family of efflux carriers comprises integral membrane proteins and has been associated with polar auxin transport during embryogenesis and endosperm development. It should be noted that no homozygous mutants were obtained for* TaDEP1*,* TaNAC2,* and* TaPIN1* by CRISPR/Cas9 editing [198] probably because they were not viable.

CRISPR/Cas9 technology was also successful in obtaining low immunogenic wheat. Sánchez-León et al. [204] have shown that CRISPR/Cas9 technology can be used to efficiently reduce the amount of alfa-gliadins in the seeds, providing bread and durum wheat lines with reduced immunoreactivity for consumers with coeliac disease. Twenty-one mutant lines were generated (15 bread wheat and 6 durum wheat), all showing a strong reduction in *α*-gliadins. Up to 35 of the 45 different genes identified in the wild-type were mutated in one of the lines, and immunoreactivity, as measured by competitive ELISA assays using two monoclonal antibodies, was reduced by 85%.

(iii) Male sterility and the induction of haploids can provide a powerful tool in cultivar breeding and genetic analysis. Generation of male-sterile and doubled haploid plants can facilitate development of hybrid seed production in wheat. In maize, the male-sterile gene 45 (*Ms45*) encodes a strictosidine synthase-like enzyme that was shown to be required for male fertility and required for exine structuring and pollen development [185]. Genetic analysis of mutated plants obtained by CRISPR/Cas9 technology demonstrated that all three wheat* Ms45 *homeologues contribute to male fertility. Mutant plants,* Tams45*-*abd*, abort pollen development resulting in male sterility.

These examples provide an insight into the many ways in which modern genome modification technologies are being used to mine the core research findings from model plant transgenesis, and finally harness that understanding to drive essential crop. The ability to enact targeted changes to the genome has revolutionized genetic modification for polyploid crop species such as wheat. There are now some enticing indications that the nearest remaining hurdle, in the form of low transformation frequencies in favoured breeding genotypes, may soon be overcome. What remains is for political entities, and the societies they represent, to adopt a more receptive view to the very real and practical benefits these technologies may provide.

## 6. Conclusions and Prospective Developments

Demand for wheat is projected to rise at a rate of 1.6% annually until 2050 as a result of increased population and prosperity. Consequently, average global wheat yields on a per hectare basis will need to increase to approximately 5 tonnes per ha from the current 3.3 tonnes [205]. Bread wheat has a very complicated hexaploid genome and, therefore, further progress in breeding of this crop is strongly dependent upon knowledge of functional genomics. Thus, it is necessary to identify the most important key-genes, their structure, role, and function in the development of wheat plants and finally for higher grain yield and better quality. Based on the knowledge of functional genomics, plant biologists can alter the structures and functions of selected key-genes through “genetic manipulation”. Genetic transformation is a very powerful tool for generating scientific proof of the roles and functions of key-genes. The authors of this review are not in a position to discuss the applications of GM-wheat in world breeding practice, since this is beyond the scope of this review. However, the term “genetic manipulation” is very broad and includes other molecular approaches that generate products that fall outside the traditional definition of “GM”. RNA interference and CRISPR/Cas9 represent modern and very advanced GM technologies that in a growing number of countries, such as USA and Canada, result in products that attract the same level of regulation as the products of traditional breeding techniques. Such “end-product-based” rather than “process-based” regulation, presents a far more favourable environment for the progression of molecular-based breeding technologies, which can and should change the future of wheat breeding across the world. However, all advanced methods will remain simply “laboratory tools” if their application is not connected with wheat breeders currently working by traditional methods. Therefore, we see the chance for real progress and positive future prospects through effective collaborations between plant molecular geneticists and wheat breeders. The application of novel methods and analysis of genetically manipulated wheat plants for their utility in breeding can be translated to the field through the introgression of genetic traits into conventional wheat breeding programs.

Awareness and concerns are also growing regarding the huge economic gaps between developed and developing countries. Underdeveloped countries are more reliant on agriculture for their overall economies, yet they have fewer opportunities to develop or collaborate on projects involving modern technologies for genetic manipulation in wheat and most other crop plants. Therefore, researchers in developed countries must take the lead and assume responsibility for sharing and freely disseminating their results and genetically manipulated wheat germplasm accessions with breeders in the developing world. This act of “research donation” can enrich lives and communities where needs are the greatest and uphold the future security and sustainability of wheat production, a key global commodity.

## Figures and Tables

**Table 1:** Comparison of parameters of agrobacterial and biolistic transformation.

Parameters	*Agrobacterium-*mediated transformation	Biolistic transformation	References
Genotype dependency	High	Less	[5]

Stability of expression of transgenes	High	High for Minimal Expression Cassettes (MEC), lower for plasmids	[6, 7]

Copy number of inserts	Around 50% single copy; Depends on the strain and transformation conditions	<50%; Depends on the amount of DNA/shot. More multicopy inserts	[7, 8]

Integration of the new genes	Random. More than one locus	Random. Often many at the same locus	[6]

Maximum transformation frequency (TF) for wheat (per 100 embryos treated)	Up to 90%	>70%	[8, 9]

Complexity of the transformation procedure in wheat	Simpler. Usually requires aseptic conditions	More complex. Requires aseptic conditions and a Biolistic Gun	[8, 10]

Main explants in wheat	Immature embryos	Immature embryos	[8, 10]

Complexity of vector construct preparation, co-transformation	More complex	Simpler	[11]

Maximum sizes of transferred inserts published	Up to 200 Kb	150–164 Kb	[12–14]

Transfer of Т-DNA borders	Yes	No (for MEC)	[15]

Transfer of vector DNA	Possible	No (for MEC); Yes (for plasmids)	[15]

Transfer of bacterial chromosomal DNA	Possible	No (for MEC)	[16]

Marker free transformation in wheat	Possible	Possible	[17–19]

*In planta* transformation in wheat	Possible	Possible	[20–22]

Delivery of RNA, proteins, nanoparticles and dyes	No	Possible	[23]

Transformation of chloroplasts and mitochondria	No	Possible	[24, 25]

Transient gene expression in different tissues and organs of plants	Efficient for limited number of plant species	Efficient	[26–28]
